# Retraction: Pemetrexed Induces S-Phase Arrest and Apoptosis via a Deregulated Activation of Akt Signaling Pathway

**DOI:** 10.1371/journal.pone.0316042

**Published:** 2024-12-13

**Authors:** The PLOS ONE Editors

After this article [1] was published, concerns were raised about results presented in Figures 1, 3, 5 and 6. Specifically:

- The following concerns were identified with the Akt and β-actin panels in Figure 1A:
  - The full panels appear similar to each other.
  - Lanes 1–4 appear similar to the Akt panel in Figure 1B.
  - Lanes 3–5 appear similar to the β-actin panel in Figure 6C.
- The β-actin panel in Figure 1B appears similar to the β-actin panel in Figure 5B.
- Lanes 2–4 of the β-actin panel in Figure 3A appear similar to the β-actin panel in Figure 5A.

In response to queries about these experiments, the first author stated that the above-listed panels in Figures 1A and 1B are incorrect, and the β-actin panel in Figure 3A is correct. They also stated that the β-actin panels in Figures 5A, 5B, 6C and 7A were included in error as these figure panels report immunoprecipitation experiments for which β-actin was not measured.

The underlying data provided was reviewed by a member of the *PLOS ONE* Editorial Board who indicated that the conclusions for Figure 5, that pemetrexed induces Cdk2 kinase activity via an Akt pathway, were not adequately explored by the experimental design in [1]. They stated that this is due to the results of the immunoprecipitation experiments which unexpectedly indicate that pemetrexed-induced Akt activity upregulates Cdk2 expression, and whether increased activity of Cdk2 was caused by its increased expression or increased phosphorylation was not experimentally addressed.

In light of the issues listed above that raise concerns about the reliability and validity of the reported results and conclusions, the *PLOS ONE* Editors retract this article.

KCC did not agree with the retraction. GCC did not respond to the final editorial decision. TYY, CCW, CCC, SLH, HWH and JWC either did not respond directly or could not be reached.
